# Insulin Receptor Substrate 2 Expression and Involvement in Neuronal Insulin Resistance in Diabetic Neuropathy

**DOI:** 10.1155/2011/212571

**Published:** 2011-06-15

**Authors:** C. W. Grote, J. K. Morris, J. M. Ryals, P. C. Geiger, D. E. Wright

**Affiliations:** ^1^Department of Anatomy and Cell Biology, The University of Kansas Medical Center, Kansas City, KS 66160, USA; ^2^Department of Molecular & Integrative Physiology, The University of Kansas Medical Center, Kansas City, KS 66160, USA

## Abstract

Insulin signaling depends on tyrosine phosphorylation of insulin receptor substrates (IRSs) to mediate downstream effects; however, elevated serine phosphorylation of IRS impairs insulin signaling. Here, we investigated IRS protein expression patterns in dorsal root ganglia (DRG) of mice and whether their signaling was affected by diabetes. Both IRS1 and IRS2 are expressed in DRG; however, IRS2 appears to be the prevalent isoform and is expressed by many DRG neuronal subtypes. Phosphorylation of Ser(731)IRS2 was significantly elevated in DRG neurons from type 1 and type 2 diabetic mice. Additionally, Akt activation and neurite outgrowth in response to insulin were significantly decreased in DRG cultures from diabetic *ob/ob* mice. These results suggest that DRG neurons express IRS proteins that are altered by diabetes similar to other peripheral tissues, and insulin signaling downstream of the insulin receptor may be impaired in sensory neurons and contribute to the pathogenesis of diabetic neuropathy.

## 1. Introduction

Diabetic neuropathy is the most common complication of diabetes, affecting 50% of patients [1]. Many patients suffer from decreased proprioception and peripheral insensitivity, causing foot injuries and falls. Alternatively, a subset of patients develop allodynia and chronic nerve pain [2]. DN is caused by a “dying back” of peripheral nerve fibers and is associated with axonal degeneration of the long axons innervating the extremities [2]. The pathology of DN is driven by multiple mechanisms, including decreased neurotrophic support [2–4], increased polyol flux [2, 3, 5], advanced glycation end products [2, 3, 5], mitochondrial dysfunction [6], and oxidative stress [2, 3, 5].

Although insulin signaling does not seem to regulate glucose uptake in neurons as it does in muscle and adipose tissue [7], insulin does appear to be crucial for proper neuron function both *in vitro* and *in vivo*. Current views now include insulin as a potent neurotrophic factor that supports neuronal growth, survival, plasticity, and maintenance of differentiated neurons [8–14]. Insulin signaling is propagated by a series of phosphorylation events that rely on a cytoplasmic docking protein known as insulin receptor substrate (IRS). Following insulin binding to the insulin receptor, intrinsic kinase activity phosphorylates tyrosine residues on both the insulin receptor and IRS. Tyrosine phosphorylation allows downstream mediators with src homology 2 (SH2) domains to bind IRS and localize to the plasma membrane. Two key SH2-containing mediators are PI3-kinase, which activates the Akt cascade, and Grb2/Sos, which activates the Ras cascade [15, 16]. These effectors drive increased transcription, translation, and translocation of proteins associated with insulin's actions.

Four mammalian isoforms of IRS have been described thus far, with IRS1 and IRS2 being the most physiologically relevant [17, 18]. IRS1 appears to be the main isoform expressed in muscle and adipose tissue [17–19], whereas IRS2 seems to be more relevant in liver and brain [17, 18, 20]. Mice with systemic IRS1 knockouts demonstrate growth retardation, insulin resistance, and beta-cell hyperplasia without diabetes [18]. IRS2 knockout mice develop insulin resistance, decreased beta-cell proliferation, overt diabetes, and female sterility [17, 18].

Serine phosphorylation of IRS has emerged as a key regulatory step for insulin signaling in both physiological and pathological situations. A large body of work suggests that elevated IRS serine phosphorylation inhibits insulin signal transduction [15, 16, 19, 21–28]. Under normal conditions, IRS is tyrosine phosphorylated upon insulin binding, but, following accumulation of activated downstream mediators, IRS is phosphorylated on serine residues, impairing insulin signaling through a negative feedback mechanism. In an insulin-resistant state, agents that promote IRS serine phosphorylation are upregulated, resulting in pathological elevation of IRS serine phosphorylation and impaired insulin signaling [15, 16, 21, 22, 27–30].

To address whether these same regulatory mechanisms affect sensory neurons, we examined IRS expression and signaling *in vivo* and *in vitro* in DRG neurons from streptozotocin- (STZ-) induced type 1 and *ob/ob* type 2 diabetic mice. Our results suggest that IRS proteins are expressed by sensory DRG neurons and undergo elevated serine phosphorylation in diabetic mice. Cultured DRG neurons from *ob/ob *animals had blunted responses to insulin as indicated by decreased Akt activation and neurite outgrowth. These findings support the hypothesis that insulin resistance due to increased serine phosphorylation of IRS2 could contribute to alterations in neuronal insulin support and promote peripheral nerve dysfunction.

## 2. Materials and Methods

### 2.1. Animals

All experiments were approved by The University of Kansas Medical Center Institutional Animal Care and Use Committee. Mice were subjected to a 12-hour light/dark cycle and had ad libitum access to food and water. For determination of IRS expression, 8-week-old male C57Bl/6 mice were purchased from Charles River (Wilmington, Mass, USA). DRG were harvested for reverse transcriptase-PCR, Western blot analysis, and immunohistochemistry. To determine the effects of diabetes on insulin signaling in peripheral neurons, male *ob/ob* mice and colony controls were ordered at 8 weeks of age from Jackson Laboratories (Bar Harbor, Maine). The *ob/ob* leptin knockout animals have been previously characterized as a model of insulin resistance, type 2 diabetes, and DN [31]. 

In addition, diabetes was induced in 8-week-old C57BL/6 male mice using a single intraperitoneal injection of streptozotocin (STZ), a pancreatic beta-cell toxin (Sigma, St. Louis, Mo), at 180 mg/kg body weight. The STZ was dissolved in 10 mM sodium citrate buffer (pH 4.5) and the nondiabetic mice were injected with 400 *μ*L of the vehicle buffer. Mice were fasted for 3 hours before and after injection to improve STZ uptake in pancreatic *β* cells. 

Body weights and blood glucose levels via tail clip were checked weekly to monitor diabetes progression. For both type 1 and type 2 models, hyperglycemia and diabetes was defined as a blood glucose level greater than 16 mM (~288 mg/dL). All STZ-injected mice with blood glucose levels below 288 mg/dL were removed from the study.

### 2.2. Adult DRG Culture

Mice were anesthetized with an intraperitoneal (i.p.) injection of cold avertin (2-2-2 Tribromoethanol) 20 *μ*L g^−1^ and transcardially perfused with ice-cold HBSS without Ca^++^/Mg^++^ (Sigma-Aldrich) to reduce protease activity and increase cell survival [32]. The DRG were harvested and transferred to 3 mL of ice-cold HBSS without Ca^++^/Mg^++^. Adult mouse DRG were cultured according to a protocol published by Malin et al. [32]. All procedures were performed in a tissue hood with appropriate sterile technique. DRG were partially digested with 2 separate enzyme solutions, one containing papain (Worthington, Lakewood, NJ, USA) and another containing collagenase type II (Worthington) and dispase type II (Roche, Basel, Switzerland). Neurons were then triturated with a fire-polished glass Pasteur pipette to dissociate neuronal cell bodies. Neurons were grown on Laminin/poly-D-lysine-coated coverslips (BD Biosciences, Bedford, MA) placed in 24-well culture plates (Sigma-Aldrich). The media consisted of insulin-free B27 supplement (Invitrogen, Carlsbad, Calif, USA), penicillin/streptomycin (Invitrogen), and F12 culture medium (Invitrogen). Neurons were then allowed to adhere to coverslips for 2 hours. After this time, either 1 mL fresh insulin-free media or media containing 100 nM insulin (Sigma-Aldrich), depending on the experimental group and assay were added. For hyperglycemia experiments, DRG neuronal cultures from wild-type C57Bl/6 mice were grown in 25 mM glucose and control cultures were maintained at 10 mM glucose based [33, 34].

### 2.3. Reverse Transcriptase Polymerase Chain Reaction

RT-PCR was performed to determine mRNA levels of different IRS isoforms. Total RNA was isolated from DRG tissue using TRI Reagent (Ambion, Foster City, CA) as indicated in the manufacturer's protocol. The RNA concentration was determined using a Bio-Rad spectrophotometer, and the quality of RNA was tested with an electrophoretic separation technique (Agilent 2100 bioanalyzer tracer with the Eukaryote Total RNA Nano Assay). RNA was then reverse transcribed to cDNA using iScript cDNA synthesis kit (Bio-Rad). The thermal cycler conditions were as follows: 25°C for 5 minutes, 42°C for 30 minutes, and 85°C for 5 minutes. Real-time PCR amplification of IRS1, IRS2, IRS3, and IRS4 was performed using 2.0 *μ*g of cDNA and SYBR green master mix (Bio-Rad). Glyceraldehyde 3-phosphate dehydrogenase (GAPDH) was used as a reference gene, and all reactions were run in triplicate. The thermal cycler conditions for PCR were 95°C and 60°C for 40 cycles. The primer sequences used for real-time PCR were as follows:

IRS1 forward: 5′-CTCTACACCCGAGACGAACAC-3′

IRS1 reverse: 5′-TGGGCCTTTGCCCGATTATG-3′

IRS2 forward: 5′-CTGCGTCCTCTCCCAAAGTG-3′

IRS2 reverse: 5′-GGGGTCATGGGCATGTAGC-3′

IRS3 forward: 5′-TCCTCCAAAGAGTGTTCCTGC-3′

IRS3 reverse: 5′-GGGGCTTGAAGTAGTCCTGC-3′

IRS4 forward: 5′-TCCTGTACCAATGCTTCTCCG-3′

IRS4 reverse: 5′-AGCTTTCTTGTTCCGACTCGT-3′

GAPDH forward: 5′-AGGTCGGTGTGAACGGAT-TTG-3′

GAPDH reverse: 5′-TGTAGACCATGTAGTTGAGG-TCA-3′

IRS isoform mRNA levels were normalized to GAPDH, and the ΔΔCt method was used for relative expression analysis.

### 2.4. Western Blots

DRG were harvested and frozen at −80°C until further use. Tissue was sonicated in Cell Extraction Buffer (CEB) (Invitrogen) containing 55.55 *μ*L/mL protease inhibitor cocktail, 200 mM Na_3_VO_4_, and 200 mM NaF. Protein from primary DRG cultures was harvested after 3 days in insulin-free media by removing growth media, adding CEB (Invitrogen), and then scraping the coverslips. Three coverslips per treatment group were combined into a single sample. The samples were incubated on ice for 1 hour with light vortexing every 10 minutes and then centrifuged at 7000 rpm for 10 minutes at 4°C. Protein concentration of the supernatant was measured with a Bradford assay (Bio-Rad, Hercules, CA). Samples were then boiled with Lane Marker Reducing Sample Buffer (Thermo Scientific, Waltham, MA) for 3 minutes. For normalization purposes, equal amounts of protein were loaded for each sample. The samples were separated on a 4–15% gradient tris-glycine gel (Bio-Rad) and then transferred to a nitrocellulose membrane. After transfer, the membrane was stained with Ponceau S and cut so that IRS (180 kDA) and Akt (60 kDA) are separated, producing 2 membranes that could be probed independently. Primary antibodies were used at the following dilutions and incubations: total IRS1 (Santa Cruz, Santa Cruz, CA) 1 : 1000 overnight at 4°C, total IRS2 (Millipore, Billerica, MA) 1 : 500 overnight at 4°C, pSer(731)IRS2 (Abcam, Cambridge, MA) 1 : 1000 overnight at 4°C, insulin receptor *β* subunit (Santa Cruz) 1 : 500 overnight at 4°C, pSer(473)Akt (Cell Signaling, Danvers, MA) 1 : 500 overnight at 4°C, total Akt (Cell Signaling) 1 : 500 overnight at 4°C, and actin (Millipore) 1 : 100,000 at room temperature for 1 hour. Antimouse and antirabbit secondary antibodies conjugated to HRP (Santa Cruz) were diluted 1 : 10,000 and incubated for 1 hour at room temperature. Band density was analyzed with ImageJ (NIH).

### 2.5. Immunohistochemistry

DRG were harvested and immediately frozen in Tissue Tek (Sakura Finetek, Torrance, CA). The tissue was sectioned at 10 *μ*m with a cryostat and placed in serial order on glass slides. Slides were blocked at room temperature for 1 hour with preincubation solution (1.5% Normal Goat or Donkey Serum, 0.5% Porcine Gelatin, 0.5% Triton X-100, and 450 *μ*L Superblock (Thermo Scientific)). Primary antibodies were used at the following dilutions and incubations: total IRS2 (Millipore) 1 : 400 overnight at 4°C, mouse monoclonal Peripherin (Millipore) 1 : 2000 overnight at 4°C, and mouse monoclonal Neurofilament 200 (Sigma) 1 : 2000 overnight at 4°C. Donkey antirabbit Alexa-488 and donkey antimouse Alexa-555 conjugated secondary antibodies (Invitrogen) were diluted 1 : 2000 and incubated for 1 hour at 4°C. Images were photographed using a Nikon Eclipse E800 microscope and analyzed with ImageJ (NIH).

### 2.6. Neurite Outgrowth

To assess potential changes in the functional neurotrophic effect of insulin on DRG neurons, neurite outgrowth was quantified in dissociated cultures of *ob/ob* mice and controls. After 5 days in culture with either insulin-free media or media containing 100 nM insulin the neurons were fixed with 4% paraformaldehyde for 10 minutes. Immunohistochemistry was performed with SMI-312 (Covance, Emeryville, CA), a pan-axonal marker, to visualize neurites and counterstained with nuclear marker, Hoechst 33342 (Invitrogen). Coverslips were mounted on slides and imaged. Neurite outgrowth area was quantified using Image J. A stereological grid was superimposed on images of the cultures, and the number of neurites crossing exactly through intersections of the grid was counted, as was the number of neuronal cell bodies producing neurites. Six coverslips per group were counted, and the neurite area per neuron was calculated according to the following equation [35]:

(1) $$\begin{array}{cc} & \frac{\left(\frac{\text{neurite}\;\text{intersections}}{\text{total}\;\text{grid}\;\text{intersections}}\right)\times \text{total}\;\text{grid}\;\text{area}}{\text{neurons}\;\text{extending}\;\text{neurites}}\\ & \;\;=\text{neurite}\;\text{area}\;\left(\mu {\text{m}}^{2}\right)\;\text{per}\;\text{neuron}. \end{array}$$

### 2.7. Statistical Analysis

All data were expressed as means ± standard error of the mean. Data were analyzed using a Student's *t*-test or 2-factor analysis of variance (ANOVA). ANOVAs were followed up with post hoc comparisons using Fisher's least significance difference where appropriate. A *P* value <  .05 was considered statistically significant.

## 3. Results

### 3.1. IRS2 Is the Predominant IRS Isoform in the DRG

**Figure 1:**
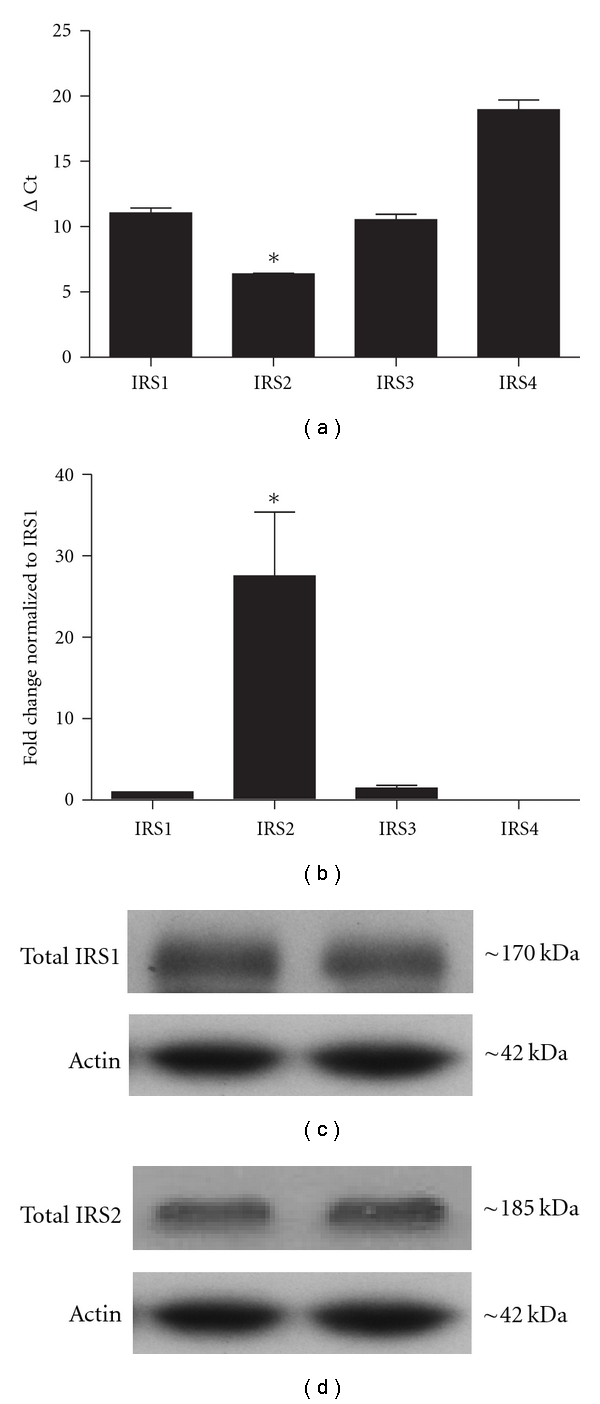
IRS isoform expression in murine lumbar DRG. IRS isoforms were examined using RT-PCR ((a) and (b)) and Western blot ((c) and (d)). (a) RT-PCR was performed on adult C57Bl/6 mouse lumbar DRG (*n* = 3 mice) and comparisons were made among IRS1, IRS2, IRS3, and IRS4. GAPDH was used as the housekeeping gene. ΔCt values for IRS2 were significantly lower than any other isoform. (b) Analysis of IRS mRNA levels that were normalized to IRS1 mRNA levels revealed that IRS2 mRNA expression was 27-fold higher than IRS1 in the DRG. *denotes *P* < .05  *n* = 3 mice. ((c) and (d)) Representative Western blots of IRS1 and IRS2 protein in mouse lumbar DRG. Equal amounts of protein (20 *μ*g) were loaded for each lane and samples were separated on 4–15% tris-glycine gel. Both IRS1 (c) and IRS2 (d) proteins were readily detectable. All mice were 8-week-old nondiabetic C57Bl/6 males.

RT-PCR was used to determine which mRNAs encoding IRS isoforms were expressed in lumbar DRG from young adult C57Bl/6 mice. Comparisons of mRNA levels for the 4 different IRS isoforms in the DRG of nondiabetic C57Bl/6 mice revealed that IRS2 mRNA is abundantly expressed within the lumbar DRG (Figures 1(a) and 1(b)). In fact, IRS2 mRNA was expressed nearly 27-fold higher relative to IRS1. In comparison, both IRS3 and IRS4 mRNAs were barely detectable in relation to IRS1 (Figures 1(a) and 1(b)). Western blots of DRG from nondiabetic C57Bl/6 mice showed that both IRS1 and IRS2 proteins were detectable in the DRG (Figures 1(c) and 1(d)). Together, these results suggest that insulin signals may be mediated through IRS substrates in the DRG and IRS2 is expressed at much higher levels similar to other neural tissues [20, 36–38].

**Figure 2:**
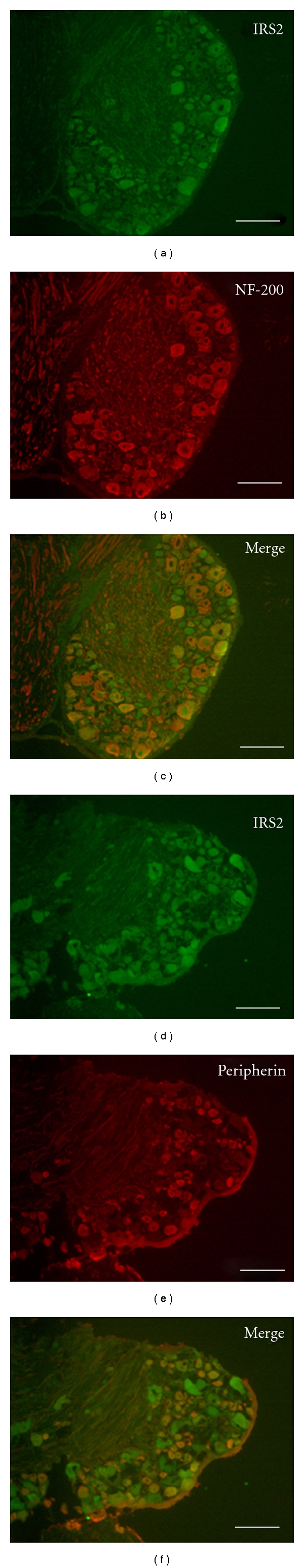
IRS2 protein expression pattern in murine lumbar DRG. Fluorescence immunohistochemistry was used to examine IRS2 expression in adult C57Bl/6 mouse lumbar DRG. ((a) and (d)) Photomicrographs of IRS2 immunoreactivity in DRG neurons. IRS2 was expressed in most neurons of the DRG in mice. ((b) and (e)) Photomicrographs of the same sections stained with antibodies to NF-200 (b), which labels large, myelinated neurons in the DRG or peripherin (e), which labels unmyelinated small DRG neurons. ((c) and (f)) Merged images of IRS2 and NF-200 (c) labeling illustrate that many IRS2-positive neurons also express NF-200, suggesting that many IRS2-positive neurons are large myelinated neurons. Similarly, merged images of IRS2 and peripherin (f) labeling illustrate that many IRS2-positive neurons coexpress peripherin, suggesting that many IRS2-positive neurons are small unmyelinated neurons. Scale bar = 100 *μ*m.

Because IRS2 was expressed at higher levels than other IRS isoforms, we focused on IRS2 expression and signaling in DRG neurons. Characterization of IRS2 expression patterns in the lumbar DRG revealed widespread IRS2 immunoreactivity throughout the ganglia (Figures 2(a) and 2(d)). IRS2-immunoreactivity was predominantly observed in the cytoplasm of neurons and not in satellite cells or Schwann cells (Figures 2(a) and 2(d)). Accordingly, identification of sensory neuron populations using antibodies to neurofilament heavy chain (NF-200, myelinated neurons, Figure 2(b)) and peripherin (unmyelinated neurons, Figure 2(e)) revealed that IRS2 was expressed by a majority of DRG neurons, and these IRS2-positive neurons included both unmyelinated and myelinated neuronal populations (Figures 2(b), 2(c), 2(e), and 2(f)).

### 3.2. Diabetes Elevates IRS2 Serine Phosphorylation in Mouse DRG

**Figure 3:**
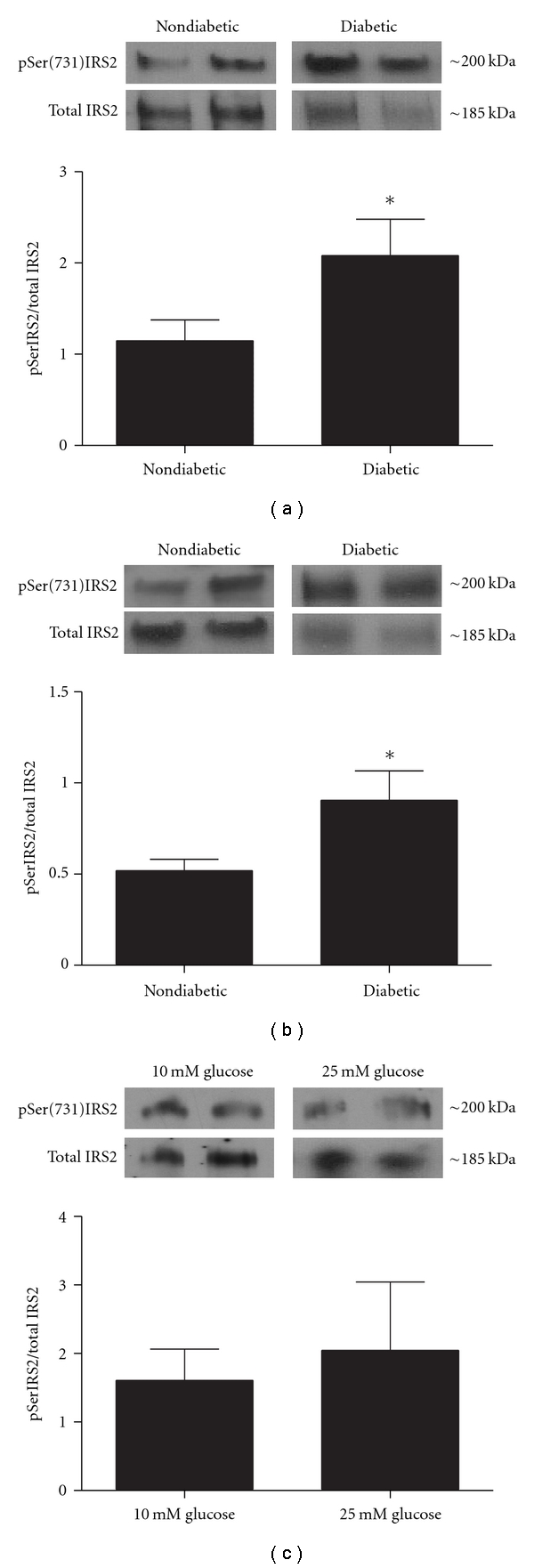
pSer(731)IRS2 is elevated in DRG neurons from type 1 and type 2 diabetic mice. Protein was harvested from adult mouse DRG culture from diabetic *ob/ob* and nondiabetic mice (a), freshly isolated DRG from STZ-injected diabetic and nondiabetic C57Bl/6 mice (b), and from DRG neurons grown in hyperglycemic and control conditions (c). Western blots were performed using antibodies that recognized phosphorylated ser731 resides on IRS2, and levels of Ser(731)IRS2 were normalized to total IRS2. (a) Comparisons of pSer(731)IRS2 levels in nondiabetic and diabetic *ob/ob* mice revealed a significant increase in pSer(731)IRS2 levels in diabetic mice. *denotes *P* < .05 versus nondiabetics. *n* = 6 for nondiabetic mice and *n* = 7 for diabetic mice. (b) Diabetes was induced in 8-week-old C57Bl/6 male mice with STZ, and diabetes was allowed to progress for 6 weeks. Similar to *ob/ob* diabetic mice, pSer(731)IRS2 levels were significantly elevated in STZ-injected diabetic mice. *denotes *P* < .05 versus nondiabetics. *n* = 5 for nondiabetic mice and *n* = 8 for diabetic mice. (c) DRG neurons from nondiabetic animals were grown in 10 mM (control) and 25 mM (hyperglycemic) glucose concentrations. There was no significant change in IRS2 serine phosphorylation levels between groups. *n* = 6 for 10 mM glucose and *n* = 7 for 25 mM glucose.

To determine whether IRS2 in the DRG is prone to diabetes-induced elevations in serine phosphorylation similar to other peripheral tissues, DRG neurons from type 2 diabetic and nondiabetic mice were cultured without insulin for 3 days and then harvested for Western blot analysis. Membranes were probed with antibodies selective for p*S*er(731)IRS2 and then stripped and probed for total IRS2 (Figure 3(a)). Quantification of p*S*er(731)IRS2 normalized to total IRS2 revealed that IRS2 serine phosphorylation was significantly increased in neurons from diabetic *ob/ob* mice compared to their nondiabetic controls (*P* < .05, Figure 3(a)). Moreover, similar elevations in p*S*er(731)IRS2 were observed in freshly harvested DRG neurons after 6 weeks of STZ-induced type 1 diabetes in C57Bl/6 mice (*P* < .05, Figure 3(b)). These results reveal that IRS2 phosphorylation of serine residues is elevated in multiple models of diabetes and, like other peripheral tissues, this serine phosphorylation could lead to suppressed insulin signaling in DRG neurons.

To investigate whether the elevated serine phosphorylation of IRS2 observed in DRG neurons harvested from diabetic mice was possibly caused by the elevated glucose levels, cultures from nondiabetic control animals were grown in hyperglycemic conditions (25 mM glucose) for 3 days and pSer(731)IRS2 levels were quantified. Results from these experiments revealed that p*S*er(731)IRS2 was not elevated in hyperglycemic cultures as compared to control cultures grown in 10 mM glucose (*P* > .05Figure 3(c)). Thus, short-term elevations in glucose levels do not appear to modify serine phosphorylation of IRS2, suggesting that the mechanisms responsible for this effect are not simply due to elevated glucose. 

**Figure 4:**
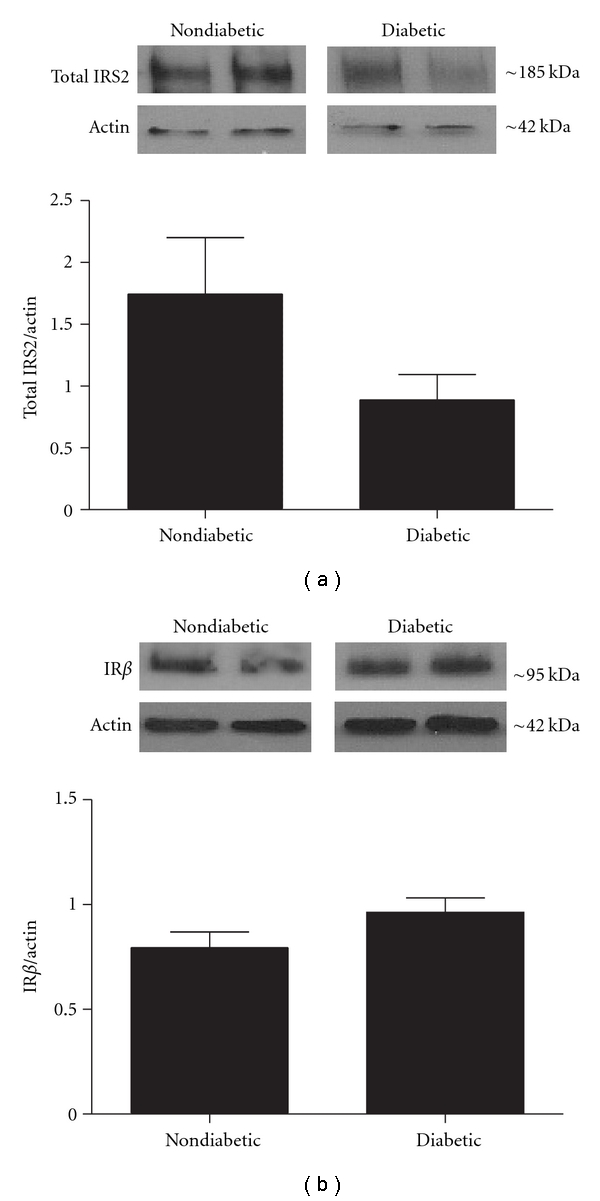
Total IRS2 and IR protein levels in mouse lumbar DRG. Protein was harvested from adult mouse DRG culture from diabetic *ob/ob* and nondiabetic mice. Western blots were performed using antibodies that recognized total IRS2 (a) or IR *β* subunit levels (b). In both cases protein levels were normalized to actin. (a) Total IRS2 levels were slightly decreased in diabetic *ob/ob* mice although this trend was not statistically significant (*P* > .05). *n* = 7 for nondiabetic mice and *n* = 7 for diabetic mice. (b) IR *β* subunit protein levels were not statistically different between diabetic and nondiabetic mice (*P* > .05). *n* = 5 for nondiabetic mice and *n* = 6 for diabetic mice.

It has been proposed that elevated serine phosphorylation of IRS proteins can lead to increased protein degradation of IRS. Thus, degradation of IRS proteins may also contribute to insulin resistance [39]. To address this possibility, we quantified total IRS2 expression in neuronal cultures from *ob/ob* diabetic mice and normalized them to the housekeeping protein actin. This analysis revealed that total IRS2 expression appeared to be decreased in DRG cultures from diabetic *ob/ob* mice although this decrease was not statistically significant (*P* > .05, Figure 4(a)). However, this finding is consistent with the view that elevated IRS2 degradation may play a role in diminished insulin signaling.

One alternative possibility for decreased insulin signaling is a downregulation of the insulin receptor (IR) in DRG of diabetic *ob/ob* mice. To examine this possibility, we measured total IR protein levels in DRG cultures from diabetic *ob/ob* mice. There were no significant differences in IR levels between DRG cultures from diabetic *ob/ob* mice and cultures from nondiabetic mice (*P* > .05, Figure 4(b)). 

### 3.3. Insulin-Stimulated Akt Activation Is Blunted in DRG Neurons from Diabetic Mice

**Figure 5:**
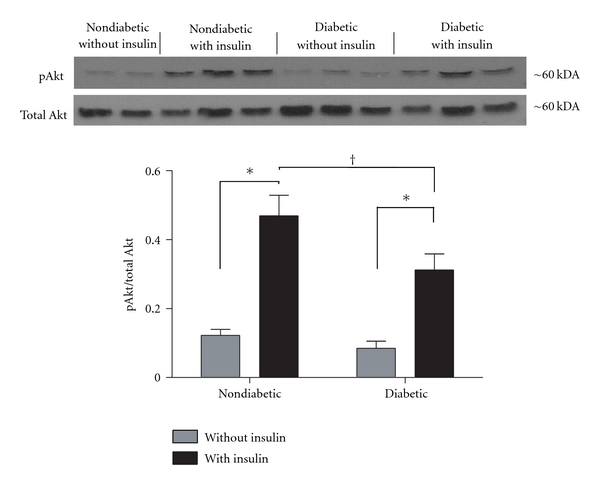
Diabetes decreases insulin-stimulated Akt activation in DRG neurons. Primary cultures of lumbar DRG neurons from *ob/ob* diabetic mice and nondiabetic controls were stimulated with 100 nM insulin for 15 minutes and harvested for Western blot. Membranes were probed for activated Akt (pSer(473)Akt) and normalized to total Akt levels. Insulin significantly increased Akt activation in both nondiabetic and diabetics cultures (*P < .05). In contrast, insulin-stimulated Akt activation in cultures from diabetic *ob/ob* mice was significantly suppressed in comparison to nondiabetic controls (^†^ *P* < .05). There were no significant differences in baseline pAkt levels between nondiabetic and diabetic mice. * and ^†^ denote *P* < .05; *n* = 5 for nondiabetic without insulin, *n* = 6 for nondiabetics with insulin, *n* = 6 for diabetics without insulin, and *n* = 6 for diabetics with insulin.

Akt is a serine kinase that is one of the major downstream mediators activated in insulin signaling in both peripheral tissues and neurons [16, 24]. In neurons, neurotrophic factor activation of the Akt pathway has been shown to promote survival and growth of neurons [40, 41]. In its activated form, Akt is phosphorylated (pAkt) on serine residue 473, and pAkt is decreased in settings of insulin resistance [42]. Here, DRG from diabetic *ob/ob* and control mice were grown in insulin-free media for 3 days and then stimulated with 100 nM insulin for 15 minutes. Cultures were then harvested for Western blot analysis to determine Akt activation in response to insulin. No significant differences were observed in baseline levels of Akt activation between diabetic and nondiabetic mice. Insulin significantly elevated activated pSer(473)Akt in cultures from both nondiabetic and diabetic mice (*P* < .05, Figure 5). However, Akt activation in response to insulin was significantly lower in cultures from diabetic *ob/ob* mice as compared to nondiabetic controls (*P* < .05, Figure 5), suggesting that the insulin signaling pathway is not being activated appropriately in the DRG of diabetic *ob/ob* mice.

### 3.4. Insulin-Stimulated Neurite Outgrowth Is Diminished in DRG Neurons from Diabetic Mice

**Figure 6:**
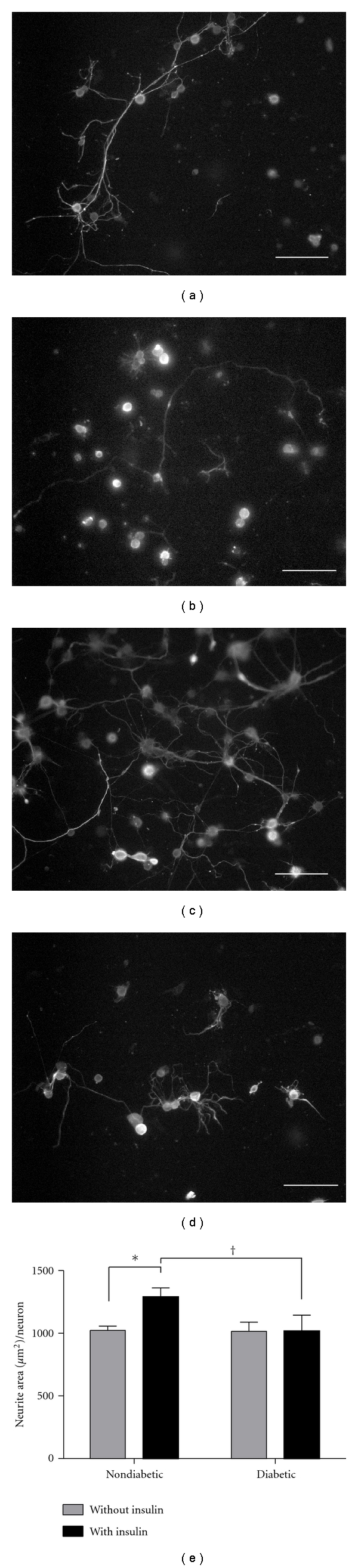
Diabetes decreases insulin-stimulated neurite outgrowth in DRG neurons. Primary cultures of lumbar DRG neurons from diabetic *ob/ob* and nondiabetic mice were grown in either insulin-free media or media containing 100 nM insulin for 5 days. Neurites were then stained with SMI 312, a pan-axonal marker. Neurite area was quantified using a stereological grid applied overtop photomicrographs of cultured wells. Photographs of representative wells are shown on the top panel: (a) nondiabetics without insulin (*n* = 5), (b) diabetics without insulin (*n* = 6), (c) nondiabetics with insulin (*n* = 6), and (d) diabetics with insulin (*n* = 5). (e) Quantification of neurite outgrowth in the different treatment groups. Insulin significantly increased neurite outgrowth in nondiabetic animals, whereas there was no effect of insulin on neurite outgrowth in neurons from diabetic mice. * and ^†^ denote *P* < .05. Scale bar = 100 *μ*m.

One common feature of neurotrophic factors is their ability to stimulate neurite outgrowth in culture. Insulin increases the percentage of neurons that produce neurites and promotes overall growth of neurites in culture [43–45]. Here, neurite outgrowth in response to insulin supplementation was used to determine whether suppressed insulin signaling correlated with alterations in neurite outgrowth. DRG cultures from diabetic *ob/ob* mice and nondiabetic controls were grown in insulin-free media or media supplemented with 100 nM insulin. After five days, the cultures were fixed and stained with SMI-312, a pan-axonal marker (Figures 6(a)–6(d)). Quantification of cultures harvested from nondiabetic and diabetic *ob/ob* mice revealed that neurite outgrowth was significantly elevated in nondiabetic cultures following insulin supplementation (*P* < .05, Figures 6(a) and 6(c)). In contrast, insulin supplementation did not affect neurite outgrowth in cultures from diabetic *ob/ob* mice. These differences suggest that cultures from diabetic *ob/ob* mice have impaired responses to insulin related to neurite outgrowth, a finding that is consistent with the hypothesis that insulin-signaling pathways in DRG neurons may be impaired by diabetes.

## 4. Discussion

The current study demonstrates that, similar to other neural tissues, IRS2 appears to be the predominant isoform of IRS in DRG neurons. Furthermore, insulin signaling in DRG neurons from diabetic mice undergoes similar modifications that have been proposed to underlie insulin resistance in adipose and muscle. These signaling alterations are consistent with blunted responses of sensory neurons to insulin stimulation, including diminished activation of downstream molecules and morphological changes. Collectively, these results provide an important step towards understanding how abnormalities in insulin signaling may impact sensory neurons and may also contribute to the development and/or progression of diabetic neuropathy. 

Although insulin resistance is a major focus in type 2 diabetes associated with adipose and muscle tissue, our understanding of the effects of reduced insulin support to sensory neurons is surprisingly lacking. A number of studies have reported expression patterns of the insulin receptor and IGF-1 receptor in DRG, but little is known about the expression of IRS isoforms in DRG. Previous studies reported that the insulin receptor is expressed primarily by small nociceptive unmyelinated neurons [46–48]. Similar expression patterns were described for the IGF-1 receptor [49], suggesting that insulin and IGF-1 may preferentially modulate small nociceptive neurons in the DRG [47]. In the current study, IRS2 protein appears to be expressed by both small and large DRG neurons, suggesting that insulin and/or IGF-1 may support a broader scope of neuronal subtypes than previously thought. Additionally, several reports have documented IRS as key docking proteins in many signaling cascades other than insulin, including neurotrophins such as brain-derived neurotrophic factor [4, 50, 51]. 

Studies are currently underway to determine if changes in IRS serine phosphorylation also lead to altered IGF1 signaling. IRS proteins are a major component of both insulin and IGF1 pathways, so it is possible that the reductions in insulin signaling through IRS serine phosphorylation may also affect proper IGF1 function. It will be important to identify these relationships, as a better understanding of how IRS proteins integrate trophic signals may shed light on mechanisms associated with neurotrophin deficiency in diabetic neuropathy [52]. Finally, although the current study focused primarily on IRS2, IRS1 was clearly detectable in the DRG. It is plausible that DRG neurons can utilize multiple IRS proteins to signal, and compensation and cooperation between IRS proteins likely exist in the DRG and should be considered in future investigations.

Schwann cell dysfunction leading to demyelination, decreased neurotrophic support, and altered protection of neurons is a known factor in the pathogenesis of DN [53]. Schwann cells express insulin receptors [54]; however, the docking protein profile has not been completely characterized. In this study, we did not observe IRS2 expression in Schwann cells, raising the possibility that insulin signaling in Schwann cells may be mediated through another IRS isoform or an additional docking protein such as, growth factor receptor-bound protein 2 (Grb2), Grb2-associated binding protein 1 (GAB1), or src homology 2 domain-containing transforming protein 1 (SHC1).

One interesting finding from this study was that IRS2 serine phosphorylation was increased in DRG from both type 1 and type 2 diabetic animals. This result was not entirely surprising as a growing body of literature has documented altered insulin responses in type 1 diabetic patients [55–58]. Most recently, Schauer et al. demonstrated that type 1 patients are insulin resistant compared to nondiabetic subjects and that the degree of insulin resistance correlated with cardiovascular disease risk [56]. This suggests that mechanisms associated with insulin resistance thought to be exclusive to type 2 diabetes may also be at work in type 1 diabetes.

In the current study, experiments were carried out *in vitro* to determine whether elevated glucose may underline the increases in serine phosphorylation of IRS2. At this point, our results do not support this view, as we saw no change in serine phosphorylation of IRS2 in neurons grown in high glucose. A failure to see insulin signaling changes in cultures exposed to abnormally high glucose levels could be a result of the short time frame, such that agents associated with hyperglycemia and increased IRS serine phosphorylation, including advanced glycation end products and reactive oxygen species, did not sufficiently alter the stress kinase activity level. Further research is required to determine the interplay of DRG IRS serine phosphorylation, hyperglycemia, and type 1 diabetes. 

If the efficacy of insulin signaling does indeed play an important role in DRG function, it is reasonable to propose that factors common to both diabetes models could play an important role in modulating IRS2 signaling regardless of available insulin levels. In that vein, chronic inflammation, increased free fatty acids, and elevated oxidative stress associated with diabetes inhibit insulin signaling by increasing serine phosphorylation of IRS1 in muscle and adipose tissue [15, 16, 21, 22, 24, 27–30, 59–61]. These various cellular stressors drive IRS serine phosphorylation by activating serine/threonine kinases. Evidence has targeted several kinases involved in this pathway, including inhibitor of kappa B kinase b (IKKb), c-Jun N-terminal kinase (JNK), mammalian target of rapamycin (mTOR), and glycogen synthase kinase-3*β* (GSK-3*β*) [16, 17, 27]. Further reports have shown that both IRS1 and IRS2 have a JNK-binding motif [16, 25] and that JNK and GSK-3*β* knockout mice display increased insulin sensitivity [60, 62]. In addition, rapamycin, an mTOR inhibitor, has been shown to improve insulin sensitivity in an *in vivo* human study and a neuronal cell line [24, 63]. Moreover, antioxidant or anti-inflammatory approaches can decrease stress kinase activity, leading to improved insulin action in muscle [42, 64]. These studies provide important links between inflammation, oxidative stress and insulin signaling, and it will be important to determine if these relationships exist and are relevant to sensory nerve dysfunction. 

Although it is likely that the inhibitory effect of IRS serine phosphorylation may be more dependent on the number of residues involved than a specific phosphorylation site [27, 65, 66], the question of how IRS serine phosphorylation may be affecting insulin signaling is not clear. Several mechanisms have been proposed, including dissociation from either the insulin receptor or plasma membrane, increased IRS proteosame degradation, interference with binding of downstream mediators, and finally, decreased tyrosine phosphorylation of the IRS protein [27]. Our finding that IRS2 appears to be slightly decreased in neuronal cultures from diabetic *ob/ob* mice is consistent with the idea that elevated serine phosphorylation can lead to IRS degradation, thus limiting the ability of insulin to stimulate downstream effectors. 

In addition to effects of insulin in the peripheral nervous system, insulin signaling in the central nervous system (CNS) is gaining considerable attention. Insulin is known to promote learning and memory [8], metabolic homeostasis [14], and have effects on aging [67]. Consequently, CNS insulin resistance has been shown to play a critical role in the development of Alzheimer's disease [68], Parkinson's disease [69], and metabolic syndrome [70]. It is now becoming evident that insulin resistance is not restricted to muscle and adipose tissue but that it also occurs in nervous tissue and can potentially be detrimental to proper neuron function.

## 5. Conclusion

The current study provides evidence that IRS proteins are expressed in the DRG and could play an important role in the ability of insulin to support peripheral neurons. Elevated serine phosphorylation of IRS proteins is a major contributing mechanism underlying insulin resistance in muscle and adipose tissue. Our results support a similar mechanism of insulin-signaling disruption within DRG neurons, and this modulatory step should be considered as an additional component contributing to diabetic neuropathy. Future studies should address mechanisms that can promote insulin sensitivity in sensory neurons, as these may be an avenue to develop therapeutic interventions that could improve sensory nerve function in both type 1 and type 2 diabetic patients.

## References

[1] Rutkove SB (2009). A 52-year-old woman with disabling peripheral neuropathy: review of diabetic polyneuropathy. *Journal of the American Medical Association*.

[2] Zochodne DW (2007). Diabetes mellitus and the peripheral nervous system: manifestations and mechanisms. *Muscle and Nerve*.

[3] Sima AAF (2003). New insights into the metabolic and molecular basis for diabetic neuropathy. *Cellular and Molecular Life Sciences*.

[4] Sullivan KA, Kim B, Feldman EL (2008). Minireview: insulin-like growth factors in the peripheral nervous system. *Endocrinology*.

[5] Tomlinson DR, Gardiner NJ (2008). Glucose neurotoxicity. *Nature Reviews Neuroscience*.

[6] Roy Chowdhury SK, Zherebitskaya E, Smith DR (2010). Mitochondrial respiratory chain dysfunction in dorsal root ganglia of streptozotocin-induced diabetic rats and its correction by insulin treatment. *Diabetes*.

[7] Patel NJ, Llewelyn JG, Wright DW, Thomas PK (1994). Glucose and leucine uptake by rat dorsal root ganglia is not insulin sensitive. *Journal of the Neurological Sciences*.

[8] McNay EC, Ong CT, McCrimmon RJ, Cresswell J, Bogan JS, Sherwin RS (2010). Hippocampal memory processes are modulated by insulin and high-fat-induced insulin resistance. *Neurobiology of Learning and Memory*.

[9] Hoybergs YMJJ, Meert TF (2007). The effect of low-dose insulin on mechanical sensitivity and allodynia in type I diabetes neuropathy. *Neuroscience Letters*.

[10] Toth C, Brussee V, Zochodne DW (2006). Remote neurotrophic support of epidermal nerve fibres in experimental diabetes. *Diabetologia*.

[11] Toth C, Brussee V, Martinez JA, McDonald D, Cunningham FA, Zochodne DW (2006). Rescue and regeneration of injured peripheral nerve axons by intrathecal insulin. *Neuroscience*.

[12] Brussee V, Cunningham FA, Zochodne DW (2004). Direct insulin signalling of neurons reverses diabetic neuropathy. *Diabetes*.

[13] Haj-ali V, Mohaddes G, Babri SH (2009). Intracerebroventricular insulin improves spatial learning and memory in male Wistar rats. *Behavioral Neuroscience*.

[14] Bruning JC, Gautam D, Burks DJ (2000). Role of brain insulin receptor in control of body weight and reproduction. *Science*.

[15] White MF (2006). Regulating insulin signaling and *β*-cell function through IRS proteins. *Canadian Journal of Physiology and Pharmacology*.

[16] White MF (2002). IRS proteins and the common path to diabetes. *American Journal of Physiology*.

[17] Sun XJ, Liu F (2009). Chapter 13 phosphorylation of IRS proteins. Yin-Yang regulation of insulin signaling. *Vitamins and Hormones*.

[18] Kahn CR (2003). Knockout mice challenge our concepts of glucose homeostasis and the pathogenesis of diabetes. *Experimental Diabesity Research*.

[19] Gupte AA, Bomhoff GL, Geiger PC (2008). Age-related differences in skeletal muscle insulin signaling: the role of stress kinases and heat shock proteins. *Journal of Applied Physiology*.

[20] Freude S, Leeser U, Müller M (2008). IRS-2 branch of IGF-1 receptor signaling is essential for appropriate timing of myelination. *Journal of Neurochemistry*.

[21] Werner ED, Lee J, Hansen L, Yuan M, Shoelson SE (2004). Insulin resistance due to phosphorylation of insulin receptor substrate-1 at serine 302. *Journal of Biological Chemistry*.

[22] Paz K, Hemi R, LeRoith D (1997). A molecular basis for insulin resistance. Elevated serine/threonine phosphorylation of IRS-1 and IRS-2 inhibits their binding to the juxtamembrane region of the insulin receptor and impairs their ability to undergo insulin-induced tyrosine phosphorylation. *Journal of Biological Chemistry*.

[23] Lee YH, Giraud J, Davis RJ, White MF (2003). c-Jun N-terminal kinase (JNK) mediates feedback inhibition of the insulin signaling cascade. *Journal of Biological Chemistry*.

[24] Mayer CM, Belsham DD (2010). Central insulin signaling is attenuated by long-term insulin exposure via insulin receptor substrate-1 serine phosphorylation, proteasomal degradation, and lysosomal insulin receptor degradation. *Endocrinology*.

[25] Aguirre V, Uchida T, Yenush L, Davis R, White MF (2000). The c-Jun NH-terminal kinase promotes insulin resistance during association with insulin receptor substrate-1 and phosphorylation of Ser(307). *Journal of Biological Chemistry*.

[26] Scioscia M, Gumaa K, Kunjara S (2006). Insulin resistance in human preeclamptic placenta is mediated by serine phosphorylation of insulin receptor substrate-1 and -2. *Journal of Clinical Endocrinology and Metabolism*.

[27] Boura-Halfon S, Zick Y (2009). Phosphorylation of IRS proteins, insulin action, and insulin resistance. *American Journal of Physiology*.

[28] Tanti JF, Jager J (2009). Cellular mechanisms of insulin resistance: role of stress-regulated serine kinases and insulin receptor substrates (IRS) serine phosphorylation. *Current Opinion in Pharmacology*.

[29] Wellen KE, Hotamisligil GS (2005). Inflammation, stress, and diabetes. *Journal of Clinical Investigation*.

[30] Thomson MJ, Williams MG, Frost SC (1997). Development of insulin resistance in 3T3-L1 adipocytes. *Journal of Biological Chemistry*.

[31] Drel VR, Mashtalir N, Ilnytska O (2006). The leptin-deficient (ob/ob) mouse: a new animal model of peripheral neuropathy of type 2 diabetes and obesity. *Diabetes*.

[32] Malin SA, Davis BM, Molliver DC (2007). Production of dissociated sensory neuron cultures and considerations for their use in studying neuronal function and plasticity. *Nature Protocols*.

[33] Zherebitskaya E, Akude E, Smith DR, Fernyhough P (2009). Development of selective axonopathy in adult sensory neurons isolated from diabetic rats: role of glucose-induced oxidative stress. *Diabetes*.

[34] Akude E, Zherebitskaya E, Chowdhury SKR, Smith DR, Dobrowsky RT, Fernyhough P (2011). Diminished superoxide generation is associated with respiratory chain dysfunction and changes in the mitochondrial proteome of sensory neurons from diabetic rats. *Diabetes*.

[35] Blacklock AD, Johnson MS, Krizsan-Agbas D, Smith PG (2005). Estrogen increases sensory nociceptor neuritogenesis in vitro by a direct, nerve growth factor-independent mechanism. *European Journal of Neuroscience*.

[36] Ye P, Li L, Lund PK, D’Ercole AJ (2002). Deficient expression of insulin receptor substrate-1 (IRS-1) fails to block insulin-like growth factor-I (IGF-I) stimulation of brain growth and myelination. *Developmental Brain Research*.

[37] Pardini AW, Nguyen HT, Figlewicz DP (2006). Distribution of insulin receptor substrate-2 in brain areas involved in energy homeostasis. *Brain Research*.

[38] Schubert M, Brazil DP, Burks DJ (2003). Insulin receptor substrate-2 deficiency impairs brain growth and promotes tau phosphorylation. *Journal of Neuroscience*.

[39] Rui L, Fisher TL, Thomas J, White MF (2001). Regulation of insulin/insulin-like growth factor-1 signaling by proteasome-mediated degradation of insulin receptor substrate-2. *Journal of Biological Chemistry*.

[40] Dudek H, Datta SR, Franke TF (1997). Regulation of neuronal survival by the serine-threonine protein kinase Akt. *Science*.

[41] Jones DM, Tucker BA, Rahimtula M, Mearow KM (2003). The synergistic effects of NGF and IGF-1 on neurite growth in adult sensory neurons: convergence on the PI 3-kinase signaling pathway. *Journal of Neurochemistry*.

[42] Gupte AA, Bomhoff GL, Morris JK, Gorres BK, Geiger PC (2009). Lipoic acid increases heat shock protein expression and inhibits stress kinase activation to improve insulin signaling in skeletal muscle from high-fat-fed rats. *Journal of Applied Physiology*.

[43] Fernyhough P, Willars GB, Lindsay RM, Tomlinson DR (1993). Insulin and insulin-like growth factor I enhance regeneration in cultured adult rat sensory neurones. *Brain Research*.

[44] Recio-Pinto E, Rechler MM, Ishii DN (1986). Effects of insulin, insulin-like growth factor-II, and nerve growth factor on neurite formation and survival cultured sympathetic and sensory neurons. *Journal of Neuroscience*.

[45] Edbladh M, Fex-Svenningsen A, Ekstrom PAR, Edstrom A (1994). Insulin and IGF-II, but not IGF-I, stimulate the in vitro regeneration of adult frog sciatic sensory axons. *Brain Research*.

[46] Sugimoto K, Murakawa Y, Sima AAF (2002). Expression and localization of insulin receptor in rat dorsal root ganglion and spinal cord. *Journal of the Peripheral Nervous System*.

[47] Baiou D, Santha P, Avelino A (2007). Neurochemical characterization of insulin receptor-expressing primary sensory neurons in wild-type and vanilloid type 1 transient receptor potential receptor knockout mice. *Journal of Comparative Neurology*.

[48] Sugimoto K, Murakawa Y, Zhang W, Xu G, Sima AAF (2000). Insulin receptor in rat peripheral nerve: its localization and alternatively spliced isoforms. *Diabetes/Metabolism Research and Reviews*.

[49] Craner MJ, Klein JP, Black JA, Waxman SG (2002). Preferential expression of IGF-I in small DRG neurons and down-regulation following injury. *NeuroReport*.

[50] Yamada M, Ohnishi H, Sano SI, Nakatani A, Ikeuchi T, Hatanaka H (1997). Insulin receptor substrate (IRS)-1 and IRS-2 are tyrosine- phosphorylated and associated with phosphatidylinositol 3-kinase in response to brain-derived neurotrophic factor in cultured cerebral cortical neurons. *Journal of Biological Chemistry*.

[51] Giovannone B, Scaldaferri ML, Federici M (2000). Insulin receptor substrate (IRS) transduction system: distinct and overlapping signaling potential. *Diabetes/Metabolism Research and Reviews*.

[52] Apfel SC (1999). Neurotrophic factors and diabetic peripheral neuropathy. *European Neurology*.

[53] Eckersley L (2002). Role of the Schwann cell in diabetic neuropathy. *International Review of Neurobiology*.

[54] Shetter AR, Muttagi G, Sagar CB (2011). Expression and localization of insulin receptors in dissociated primary cultures of rat Schwann cells. *Cell Biology International*.

[55] Kilpatrick ES, Rigby AS, Atkin SL (2007). Insulin resistance, the metabolic syndrome, and complication risk in type 1 diabetes: "double diabetes" in the diabetes control and complications trial. *Diabetes Care*.

[56] Schauer IE, Snell-Bergeon JK, Bergman BC (2011). Insulin resistance, defective insulin-mediated fatty acid suppression, and coronary artery calcification in subjects with and without type 1 diabetes the CACTI study. *Diabetes*.

[57] DeFronzo RA, Hendler R, Simonson D (1982). Insulin resistance is a prominent feature of insulin-dependent diabetes. *Diabetes*.

[58] Orchard TJ, Olson JC, Erbey JR (2003). Insulin resistance-related factors, but not glycemia, predict coronary artery disease in type 1 diabetes: 10-year follow-up data from the Pittsburgh Epidemiology of Diabetes Complications Study. *Diabetes Care*.

[59] Ueno M, Carvalheira JBC, Tambascia RC (2005). Regulation of insulin signalling by hyperinsulinaemia: role of IRS-1/2 serine phosphorylation and the mTOR/p70 S6K pathway. *Diabetologia*.

[60] Hirosumi J, Tuncman G, Chang L (2002). A central, role for JNK in obesity and insulin resistance. *Nature*.

[61] Zick Y (2005). Ser/Thr phosphorylation of IRS proteins: a molecular basis for insulin resistance. *Science’s STKE*.

[62] MacAulay K, Doble BW, Patel S (2007). Glycogen synthase kinase 3alpha-specific regulation of murine hepatic glycogen metabolism. *Cell Metabolism*.

[63] Krebs M, Brunmair B, Brehm A (2007). The mammalian target of rapamycin pathway regulates nutrient-sensitive glucose uptake in man. *Diabetes*.

[64] Jiang G, Dallas-Yang Q, Liu F, Moller DE, Zhang BB (2003). Salicylic acid reverses phorbol 12-myristate-13-acetate (PMA)- and tumor necrosis factor *α* (TNF*α*)-induced insulin receptor substrate 1 (IRS1) serine 307 phosphorylation and insulin resistance in human embryonic kidney 293 (HEK293) cells. *Journal of Biological Chemistry*.

[65] Herschkovitz A, Liu YF, Ilan E, Ronen D, Boura-Halfon S, Zick Y (2007). Common inhibitory serine sites phosphorylated by IRS-1 kinases, triggered by insulin and inducers of insulin resistance. *Journal of Biological Chemistry*.

[66] Liu YF, Herschkovitz A, Boura-Halfon S (2004). Serine phosphorylation proximal to its phosphotyrosine binding domain inhibits insulin receptor substrate 1 function and promotes insulin resistance. *Molecular and Cellular Biology*.

[67] Broughton S, Partridge L (2009). Insulin/IGF-like signalling, the central nervous system and aging. *Biochemical Journal*.

[68] Sima AAF (2010). Encephalopathies: the emerging diabetic complications. *Acta Diabetologica*.

[69] Morris JK, Bomhoff GL, Stanford JA, Geiger PC (2010). Neurodegeneration in an animal model of Parkinson's disease is exacerbated by a high-fat diet. *American Journal of Physiology*.

[70] Gerozissis K (2008). Brain insulin, energy and glucose homeostasis; genes, environment and metabolic pathologies. *European Journal of Pharmacology*.

